# Differentiation therapy: sesamin as an effective agent in targeting cancer stem-like side population cells of human gallbladder carcinoma

**DOI:** 10.1186/1472-6882-14-254

**Published:** 2014-07-19

**Authors:** Xiang Kong, Ming-zhe Ma, Yan Zhang, Ming-zhe Weng, Wei Gong, Li-qun Guo, Jun-xiu Zhang, Guo-dong Wang, Qing Su, Zhi-wei Quan, Jie-ren Yang

**Affiliations:** 1 Department of Endocrinology, Xinhua Hospital, Shanghai Jiaotong University School of Medicine, Shanghai 200092, China; 2 Department of Pharmacology, Wannan Medical College, Wuhu, Anhui 241002, China; 3 Department of General Surgery, Xinhua Hospital, Shanghai Jiaotong University School of Medicine, Shanghai 200092, China; 4 Department of Gastroenterology, Yijishan Hospital Affiliated to Wannan Medical College, Wuhu, Anhui 241001, China; 5 Department of Pharmacy, Wannan Medical College, Wuhu, Anhui 241002, China

## Abstract

**Background:**

Recent studies have demonstrated that side population (SP) cells isolated from various cancer cell lines and primary tumors possess stem cell-like properties. Sesamin, a food-derived agent, possesses anti-cancer activities both *in vitro* and *in vivo*. The present study was designed to determine whether sesamin also have effects on cancer stem-like SP cells from gallbladder cancer (GBC).

**Methods:**

In this study, we sorted SP cells by flow cytometry. SP cells were cultured and treated with sesamin. Tumor-sphere formation, colony formation, Matrigel invasion and tumorigenic potential were determined. Expression of nuclear NF-κB, IL-6, p-Stat3, Twist, E-cadherin and Vimentin was measured by Western blot, immunofluorescence staining or RT-PCR analysis. Nuclear NF-κB activity and IL-6 protein level were assessed with ELISA. Xenograft tumors were generated in nude mice.

**Results:**

After treated with sesamin, SP cells differentiated into cells expressing the epithelial marker (E-cadherin). Sesamin effectively affected SP cells stem cell-like characteristics (i.e., tumor-sphere formation, colony-formation, Matrigel invasion), weakened the drug-resistance of SP cells and inhibited tumor growth both *in vitro* and *in vivo*. Treatment with sesamin significantly reduced the expression of nuclear NF-κB, IL-6, p-Stat3, Twist and Vimentin (a mesenchymal marker) in SP cells. Nuclear NF-κB activity and IL-6 level were also decreased after treatment with sesamin.

**Conclusion:**

Food-derived sesamin directs the epithelial differentiation of cancer stem-like SP cells from GBC, which is associated with attenuation of NF-κB-IL-6-Stat3-Twist signal pathway.

## Background

Gallbladder cancer (GBC) is the most common biliary tract cancer and the fifth most common gastrointestinal malignancy [1]. The outcome of patients with more advanced disease is dismal with 5-year survival rates ranging from 20% to 40% [2]. Despite advances in chemo-radiation and adjuvant chemotherapy, chemotherapeutic options prolong life minimally as chemo-resistance is one of the most remarkable characteristics of GBC [3].

The concepts of cancer stem cells (CSCs) or tumor-initiating cells have proposed that the heterogeneous tumor cell population contains a small population of cells with properties such as self-renewal, multiplex differentiation, chemo- and radio-resistance, high tumorigenicity, and they may play pivotal parts in the development, progression, metastasis, recurrence and multidrug resistance of cancer [4,5]. In this study, we employed the side-population (SP) cells, which are enriched in a subset of cancer stem-like cells and are considered as CSCs in a variety of cancer [6-9].

Sesamin is isolated from the oil of sesame seeds and exerts a variety of biological activities, including lipid-lowering, antihypertensive and antitumor effects [10-12]. Regarding its antitumor effects, sesamin has been demonstrated to inhibit the growth of a variety of cancer cells both *in vivo* and *in vitro*, including breast cancer [13,14], human lung cancer [15] and colon cancer [16]. The mechanisms by which sesamin exerts antitumor effects are not fully understood, but its role in suppression of NF-κB and interleukin 6 (IL-6) have been clarified [12,17]. In fact, NF-κB and IL-6 is vital in the epigenetic switch from immortalized breast cells to a stably transformed line that contains CSCs [18]. IL-6 promoted E-cadherin repression, which has been implicated in the generation of a stem cell phenotype [19,20]. And besides, accumulating evidences have linked epithelial-mesenchymal transition (EMT) to CSCs. Down-regulation of E-cadherin generates a mesenchymal phenotype, which displays stem cell-like characteristics [21]. We therefore investigated whether sesamin could modify the stem cell-like characteristics of SP cells through inducing the epithelial differentiation. We further explored the underlying mechanisms of the effects that sesamin exerts on SP cells of GBC.

## Methods

### Ethics statement

All animal experiments were performed in animal laboratory center of Xinhua Hospital and in accordance with the Guide for the Care and Use of Laboratory Animals published by the US National Institutes of Health (NIH publication no. 85–23, revised 1996). The study protocol was approved by the Animal Care and Use committee of Xinhua Hospital.

### Cell culture

Two human GBC cell lines were used in the experiment. SGC-996 and GBC-SD were purchased from Cell Bank of the Chinese Academy of Science (Shanghai, China). SGC-996 and GBC-SD cell lines were cultured in Dulbecco’s modified Eagle’s medium (DMEM, Gibco BRL), containg 10% fetal bovine serum (FBS, HyClone) as well as 100U/ml penicillin and 100 μg/ml streptomycin. Cells were maintained at 37°C in 5% CO_2_. Sesamin (>94% purity) was provided by Tianyi Lvbao Technology Co. (Wuhu, China) [11]. Sesamin was dissolved in DMSO as 0, 11, 33.3, 100 μM stock solution. Vehicle control consisted of DMSO equivalent to treatments.

### Flow cytometry analyses

To sort the SP cells from GBC cell lines, cells were trypsinized in a logarithmic growth phase and washed with DMEM containing 2% FBS and 10 mmol/L of HEPES twice. For each SP analysis, cells (1 × 10^6^ cells/mL) were incubated in pre-warmed DMEM with 2% FBS containing freshly added Hoechst 33342 (5 μg/mL final concentration) in the presence or absence of 50 μg/ml verapamil (Sigma-Aldrich) for 90 minutes at 37°C in water bath. During the incubation time, cells were protected from light and mixed by gentle vortexing every 15 minutes. At the end of the incubation, samples were washed with Hank’s Balanced Salt Solution supplemented with 2% FBS and 10 mmol/L of HEPES and re-suspended at a final concentration of 1 × 10^6^ cells/mL. Before running samples on a flow cytometer (Becton Dickinson), propidium iodide was added to a final concentration of 1 μg/mL to exclude dead cells. Hoechst 33342 was excited with an ultraviolet laser at 350 nm, and fluorescence emission was measured with DF 424/44 (Hoechst blue) and DF 630/22 (Hoechst red) optical filters.

To determine the multi-differentiation capacity of SP cells, cells were cultured under differentiating conditions (DMEM supplemented with 10% FBS in the absence of growth factors). Cells were retained with Hoechst dye at 3 and 7 days, and the fraction of SP cells was analyzed with flow cytometer.

To determine the effects of sesamin on SP cells population, the sorted SP cells were given various concentrations of sesamin (0, 11, 33.3, 100 μM) for 7 days in un-differentiating conditions: DMEM/F12 medium (Gibco BRL) supplemented with 20 ng/mL human recombinant epidermal growth factor (EGF; Invitrogen) and 10 ng/mL human recombinant basic fibroblast growth factor (bFGF; Invitrogen), as well as 100U/ml penicillin and 100 μg/ml streptomycin. The fraction of SP cells was analyzed by flow cytometry. Cells without sesamin treatment were set as control group.

### *In vitro* propagation of SP cells and tumor-sphere assay

For *in vitro* propagation, the sorted SP cells were plated on ultralow attachment six well plates (Sigma-Aldrich) at a density of 2 × 10^4^ cells/mL in un-differentiating conditions: DMEM/F12 medium supplemented with 20 ng/mL EGF and 10 ng/mL bFGF. Additional 0.5 mL of medium was added every two days. Cell aggregates known as tumor-spheres were formed within 3 days after seeding.

To test the tumor-sphere formation ability, SP cells and non-SP cells were seeded at 5 × 10^3^ cells/mL in DMEM/F12 medium supplemented with 20 ng/mL EGF and 10 ng/mL bFGF. After 7 days, the formed tumor-spheres derived from SP cells were collected, trypsinized into single-cell suspensions and re-cultured in DMEM/F12 medium supplemented with 20 ng/mL EGF and 10 ng/mL bFGF to form secondary tumor-spheres. After exposing to sesamin at various concentrations (0, 11, 33.3, 100 μM) in DMEM/F12 medium supplemented with 20 ng/mL EGF and 10 ng/mL bFGF for 7 days, the number of tumor-spheres formed were observed and counted utilizing a Leica DC 200 microscope. The control group was without treatment with sesamin.

### Colony formation assay

To examine clonogenic ability, non-SP cells, SP cells and SP cells pretreated with sesamin of various concentrations (0, 11, 33, 100 μM) for 7 days were seeded in six-well plates at a density of 200 cells/well and maintained in DMEM with 10% FBS. Cells were washed with phosphate buffered saline (PBS), fixed in methanol for 15 minutes and stained with 0.5% crystal violet for 15 minutes. The plates were then photographed, and the colonies were counted.

### Matrigel invasion assay

Inserts with 8 μM pore (Millipore) were pre-coated with matrigel (BD Biosciences) at a concentration of 3 mg/mL for 3 hours. Non-SP cells, SP cells and SP cells pretreated with sesamin of various concentrations (0, 11, 33, 100 μM) for 7 days at a density of 1 × 10^4^ viable cells in 200 μl of serum-free DMEM medium of each permutation were added to their respective upper chamber, DMEM + 10% FBS was placed in the lower compartments as chemo-attractants. The plates were incubated for 24 hours at 37°C in 5% CO_2_ atmosphere. At the end of incubation, cells that did not migrate or invade through the pores were removed by a cotton swab. Cells on the lower surface were fixed in ice-cold 100% methanol, stained in 0.5% crystal violet and scored as the mean number of invaded cells per 5 random optical fields at 20 × magnification.

### Immunofluorescence microscopy

For membrane staining (E-cadherin), cells were fixed by incubation with cold 100% methanol for 10 minutes. For intracellular staining (Vimentin), the cells were fixed with 4% (wt/vol) paraformaldehyde in PBS and permeabilized by incubation with 0.5% Triton X-100 in PBS for 1 minute. The cells were incubated with 3% bovine serum albumin in PBS for 30 minutes at room temperature. After washing with PBS, the cells were incubated with specific primary antibody at 4°C overnight. The cells were then washed and incubated with Alexa Fluor 488- or 555-conjugated goat anti-rabbit IgG diluted in blocking solutions and incubated for 1 hour. The nuclei were stained with 4,6-diamidino-2-phenylindole (DAPI). Sections were visualized by fluorescence microscopy. SP cells were cultured under differentiating conditions (DMEM supplemented with 10% FBS in the absence of growth factors) for 7 days to allow cells attachment and differentiation. In addition, SP cells were treated with 100 μM sesamin for 7 days in DMEM/F12 medium supplemented with 20 ng/mL EGF and 10 ng/mL bFGF. The acquisition of epithelial markers (E-cadherin) and loss of mesenchymal markers (Vimentin) were evaluated by immunofluorescence as indicated above.

### Cell proliferation assay

Cell proliferation assays were conducted using the CCK-8 assay kits as described by the manufacturer. Sorted SP cells and non-SP cells were cultured in 96-well plates for 3 days in DMEM/F12 medium supplemented with 20 ng/mL EGF and 10 ng/mL bFGF. For the chemo-resistance of SP cells, the same amount of SP and non-SP cells were treated with cisplatin at a range of concentrations (0, 2, 4, 8, 16 μM) for 96 hours in DMEM/F12 medium supplemented with 20 ng/mL EGF and 10 ng/mL bFGF. Treatment with sesamin at a variety of concentrations (0, 11, 33.3, 100 μM) for 3 and 7 days in DMEM/F12 medium supplemented with 20 ng/mL EGF and 10 ng/mL bFGF was performed to test the tumor-inhibition effects on SP cells. For the chemosensitization effects of sesamin on SP cells, sesamin alone (33.3 μM), cisplatin alone (4 μM), sesamin plus cisplatin (33.3 plus 4 μM) were added to respective wells for an incubation of 7 days.

### IL-6 ELISA assay

Sorted SP cells and non-SP cells were cultured in 96-well plates at a density of 2 × 10^4^ cells/mL in DMEM/F12 medium supplemented with 20 ng/mL EGF and 10 ng/mL bFGF. Conditioned medium was collected over 48 hours and the IL-6 concentrations were tested utilizing the human IL-6 ELISA Development Kit (Peprotech) according to the manufacturer’s instructions. Briefly, culture medium and IL-6 standards were incubated for 2 hours at room temperature in 96-well microplates, which were coated with IL-6 mAb. After washing, an antibody against IL-6 conjugated to alkaline phosphatase was added. Substrate and amplifier were added and the plates were read at 485 nm. Similar procedures were performed to study the effects of sesamin (0, 11, 33.3, 100 μM) for 7 days on IL-6 production.

### Real-time reverse transcriptase PCR analysis

For non-SP cells, SP cells and SP cells exposed to sesamin (0, 11, 33.3, 100 μM) for 7 days, total cellular RNA were collected from cells using 1 mL of TRIzol reagent (Invitrogen), then the samples were reverse-transcribed using random hexamers and reverse transcriptase (Invitrogen) to obtain cDNA. The expression levels of IL-6 mRNAs were determined by real-time reverse transcriptase-PCR. All reactions were carried out on 96-well PCR plates (ABI PRISM, Applied Biosystems) in an ABI PRISM 7000 sequence detection system. Standard thermal cycling conditions are a hot start at 50°C for 5 min, 95°C 10 min, followed by up to 50 cycles of: 95°C 15 sec, 60°C for 1 min. Data shown are normalized to GADPH expression. Primer sequences were as follows:

IL-6, 5′-GAGAAAGGAGACATGTAACAAGAGT-3′ (forward), 5′-GCGCAGAATGAGATGAGTTGT-3′ (reverse);

GADPH, 5-TGGGGAAGGTGAAGGTCGG-3′ (forward), 5′-CTGGAAGATGGTGATGGGA- 3′ (reverse).

### Western blot analysis

For non-SP cells, SP cells and SP cells exposed to sesamin (0, 11, 33.3, 100 μM) for 7 days, nuclear and cytosolic proteins were prepared using NE-PER Nuclear and Cytoplasmic Fractions Kit purchased from Thermo Scientific. Protein content was determined by Bradford assay. Equal amounts (30–50 μg) of proteins were applied to an 8-12% SDS-polyacrylamide separating gel and transferred to a PVDF membrane (Millipore). The membrane was blocked with 5% skim milk or 1% BSA in TBST and then probed with indicated primary antibodies with gentle shaking at 4°C overnight. Primary antibodies against NF-κB (p65, 1:1000), E-cadherin (1:400), Vimentin (1:500), Twist (1:400) (Abcam), signal transducer and activator of transcription 3 (Stat3, 1:1000) and p-Stat3 (Tyr-705, 1:800) (Cell Signaling Technology) were used in this study. After washing the membranes three times, the immunoblots were incubated with the appropriated secondary antibodies for 1 hour. Antibody-bound proteins were detected by Millipore enhanced chemiluminescence kit.

### NF-κB/p65 activity assay

The sorted SP cells were plated on ultralow attachment six well plates at a density of 2 × 10^4^ cells/mL in un-differentiating conditions: DMEM/F12 medium supplemented with 20 ng/mL EGF and 10 ng/mL bFGF. The sorted SP cells were treatment with sesamin at a variety of concentrations (0, 11, 33.3, 100 μM) for 7 days. Resuspend cell pellet in 0.5 ml of ice cold Hypotonic Buffer (20 mM Tris–HCl, 10 mM NaCl, 1 mM EDTA, 2 mM Na3VO4, containing protease inhibitors) by pipetting up and down several times and transfer to a pre-chilled microcentrifuge tube. The cells were then lysed with 15 μl of 10% nonyl phenoxylpolyethoxylethanol (NP-40). The nuclear pellet was resuspended in 30 μl of Nuclear Lysis Buffer (Imgenex). Vortex vigorously and incubate suspension for 30 min on a rocking platform. Then, the extract was centrifuged, and the supernatant containing the nuclear extract was obtained.

NF-κB activity was measured by a NF-κB p65 ACTIVELISA kit (Imgenex) according the manufacturer’s instruction. Briefly, Free p65 was captured by the anti-p65 antibody coated plate, then after adding a second anti-p65 antibody followed by alkaline phosphatase-conjugated secondary antibody the amount of bound p65 is detected using colorimetric detection in an ELISA plate reader at absorbance 405 nm.

### In vivo tumor growth assay

1 × 10^2^ to 1 × 10^6^ cells/100 μL PBS of non-SP and SP cells were injected subcutaneously into 6-week-old nude mice (n = 4 per group). Caliper measurement of tumor volume (length × width) was conducted every week. Tumor volumes were calculated with the formula: length (mm) × width (mm)^2^/2. In addition, after pretreated with sesamin (100 μM) for 7 days, 1 × 10^5^ SP cells were injected subcutaneously into 6-week-old nude mice (n = 6 each group). Tumor volumes were measured weekly.

### Statistical analysis

Each experiment was carried out in duplicate and at least three independent experiments were done. Data were shown as mean ± SD. Comparisons among groups were determined by the use of ANOVA followed by a Newman–Keuls test. The difference was considered statistically significant when *p* < 0.05.

## Results

### Sorting the SP cells of GBC

**Figure 1:**
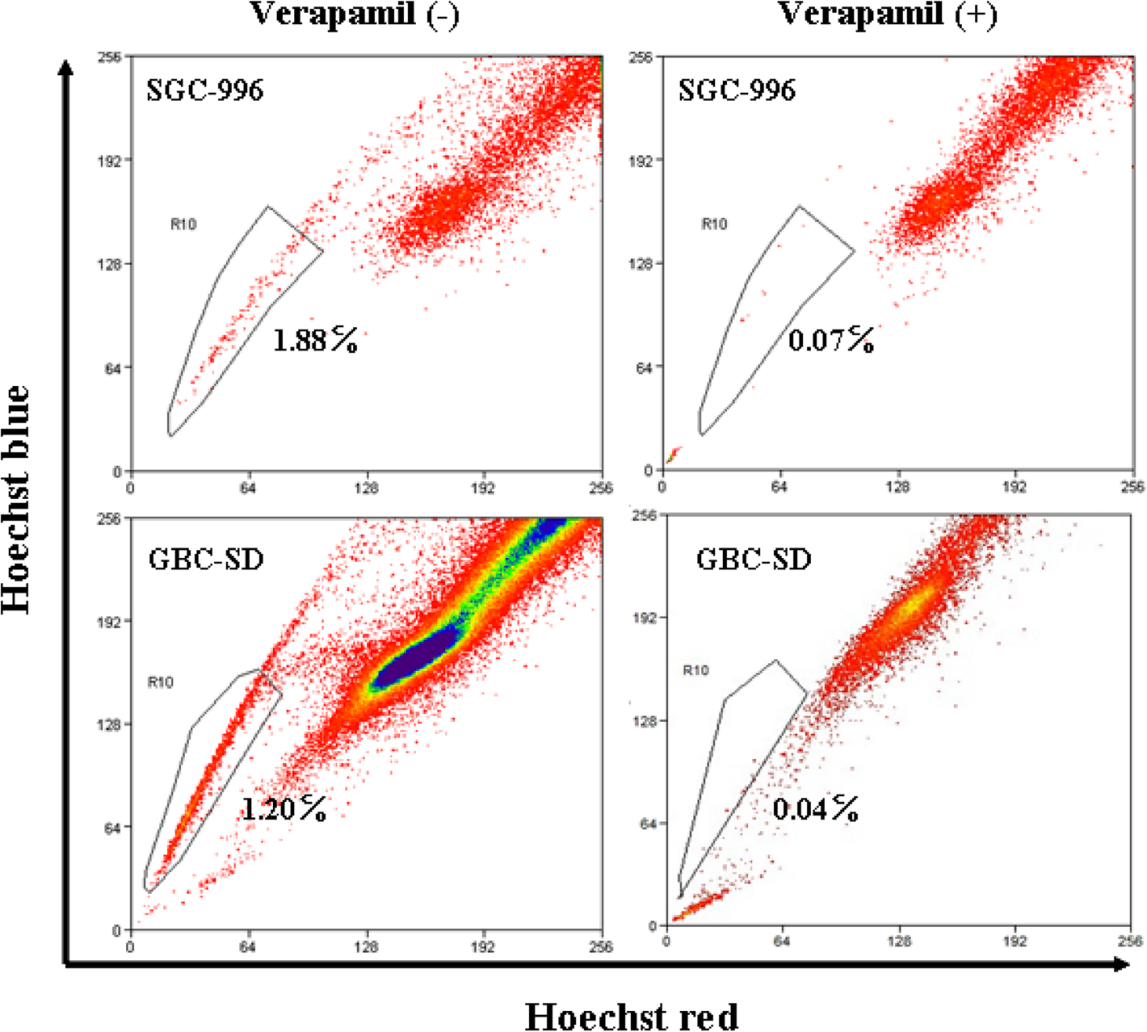
**SP cells analysis.** The proportion of SP cells in SGC-996 and GBC-SD was analyzed by flow cytomertry. As a control, verapamil (50 μM) was added to inhibit the dye efflux activity.

The proportion of SP cells were 1.85 ± 0.31% and 1.14 ± 0.23% obtained from SGC-996 and GBC-SD cell lines respectively, which decreased significantly in the presence of verapamil (Figure 1). Our sorting of SP cells was based on the ability to efflux the dye Hoechst 33342. Verapamil was used as a control to effectively block the efflux of the Hoechst dye, which reveals that this method of sorting SP cells is effective.

### Biological characteristics of cancer stem-like SP cells

**Figure 2:**
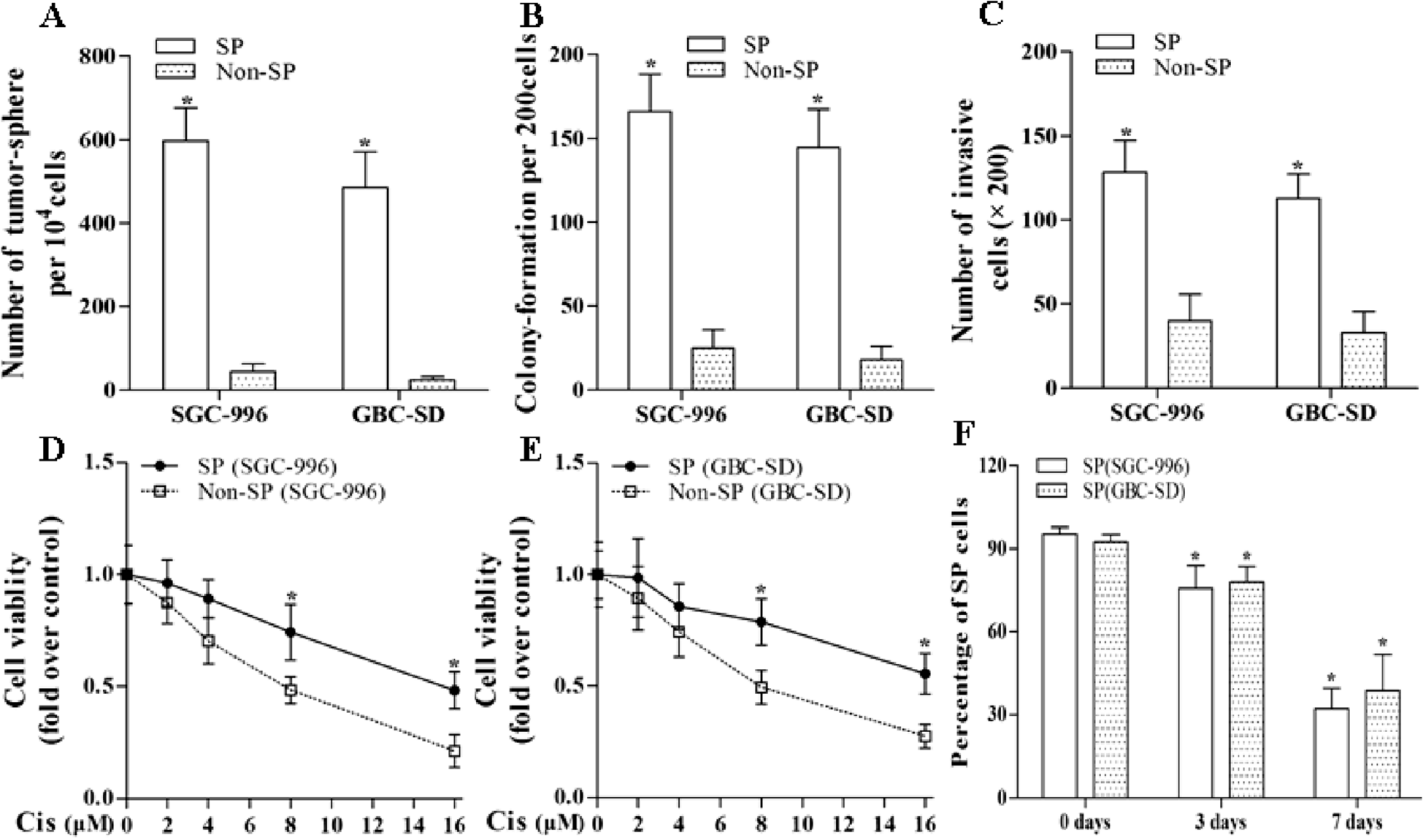
**Biological characteristics of cancer stem-like SP cells.** SP cells had higher tumor-sphere formation ability than the non-SP cells under un-differentiating conditions (DMEM/F12 medium supplemented with 20 ng/mL EGF and 10 ng/mL bFGF) **(A)**. SP cells demonstrated much higher colony formation ability **(B)** and invasion capacity **(C)**. SP cells were more resistant to cisplatin (Cis) compared with non-SP cells **(D** and **E)** SP cells fraction decreased gradually under differentiating conditions (DMEM supplemented with 10% FBS) **(F)**. *p < 0.05 *vs.* non-SP cells group or control group.

Self-renewal, multi-differentiation capacity, tumor initiation are proposed to be characteristics of CSCs [4]. Therefore, we determined the stem cell-like capacity of the SP cells both *in vitro* and *in vivo*. The cell viabilities of SP cells obtained from SGC-996 and GBC-SD were increased by 44% and 49% respectively compared to those in non-SP cells at day 3. In terms of self-renewal, SP cells had 13- and 20-fold higher tumor-sphere formation ability than the non-SP cells under un-differentiating conditions (Figure 2A). SP cells also demonstrated much higher colony formation ability compared with non-SP cells (Figure 2B). Furthermore, SP cells had elevated invasion capacity (Figure 2C). As shown in Figure 2D and E, SP cells were more chemo-resistant to the conventional drug cisplatin compared with non-SP cells.To determine the multi-differentiation capacity of SP cells, sorted SP and non-SP cells were cultured in collagen-coated dishes under differentiating conditions. Our data indicated that the SP faction rapidly underwent a symmetric division and generated both SP and non-SP cells, namely the SP enriched population went down by approximately 66%, 58% from SGC-996 and GBC-SD cell lines respectively (Figure 2F). In sharp contrast, the sorted non-SP cells were not able to generated SP cells in this period of time (data not shown).

In the *in vivo* xenograft experiments, we found that the injection of SP cells, as few as 10^2^ cells per mouse, was able to generate tumor xenografts, while 10^4^ non-SP cells were much less tumorigenic (Table 1). In all, the SP cells of GBC were in accordance with the characteristics of CSCs. Therefore, we considered the SP cells as CSCs.

**Table 1:** **Tumor incidence of SP cells and non-SP cells *in vivo***

**Cell population**	**10**^**6**^	**10**^**5**^	**10**^**4**^	**10**^**3**^	**10**^**2**^
	**Tumors**	**Tumors**	**Tumors**	**Tumors**	**Tumors**
SP(SGC-996)	4/4	4/4	3/4	2/4	2/4
Non-SP(SGC-996)	4/4	2/4	1/4	0/4	0/4
SP(GBC-SD)	4/4	4/4	4/4	2/4	2/4
Non-SP(GBC-SD)	4/4	3/4	2/4	0/4	0/4

Increased tumorigenicity of SP cells when injected subcutaneously into nude mice (n = 4 per group). The observational period lasted for six weeks.

### Molecular characteristics of SP Cells

**Figure 3:**
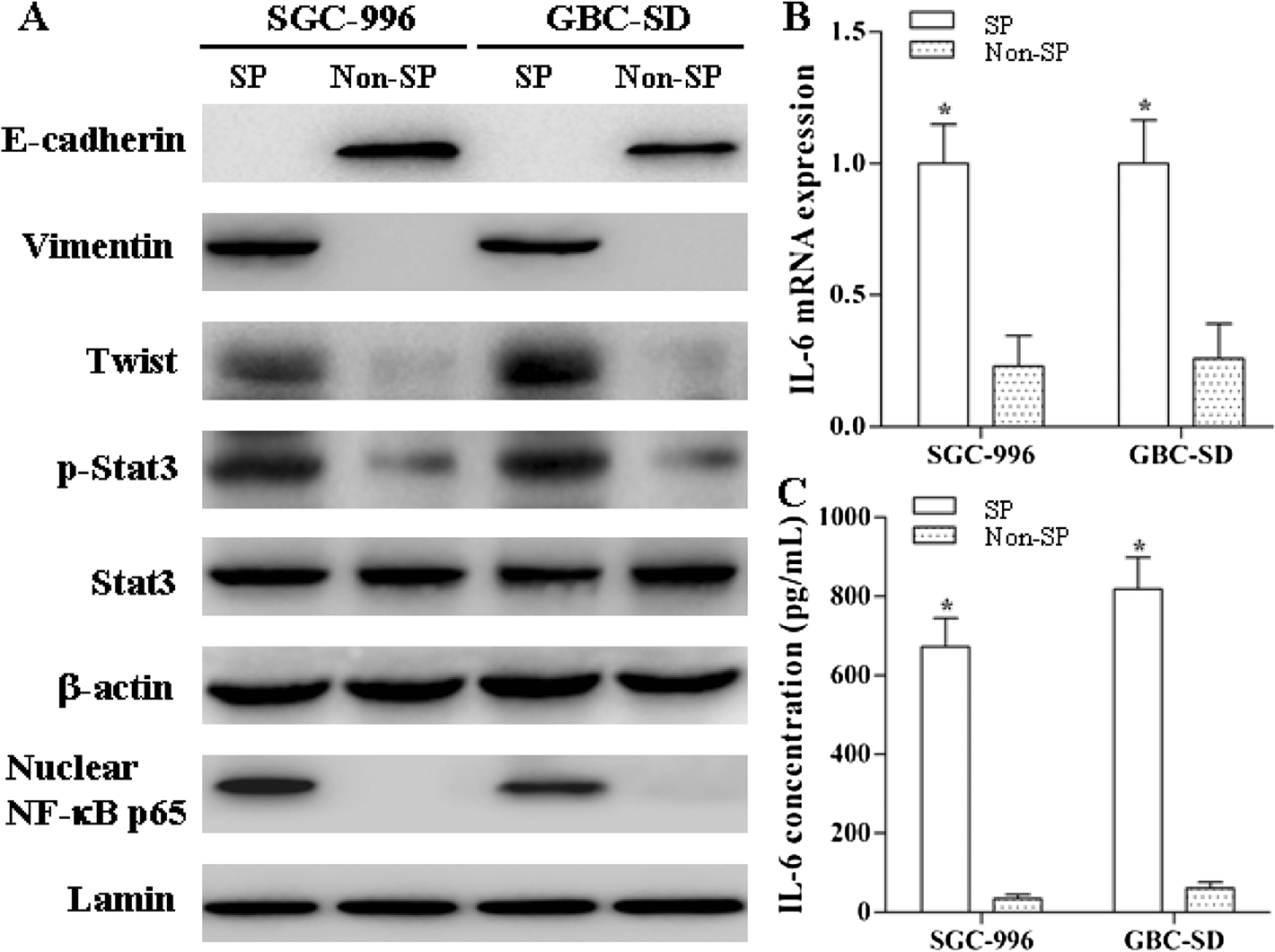
**Molecular characteristics of SP cells.** The protein expression of E-cadherin, Vimentin, Twist, Stat3, p-Stat3 and nuclear NF-κB was detected by Western blot analysis **(A)**. IL-6 mRNA and protein level were measured by real-time reverse transcriptase PCR and ELISA respectively **(B** and **C)**. *p < 0.05 *vs.* non-SP cells group.

**Figure 4:**
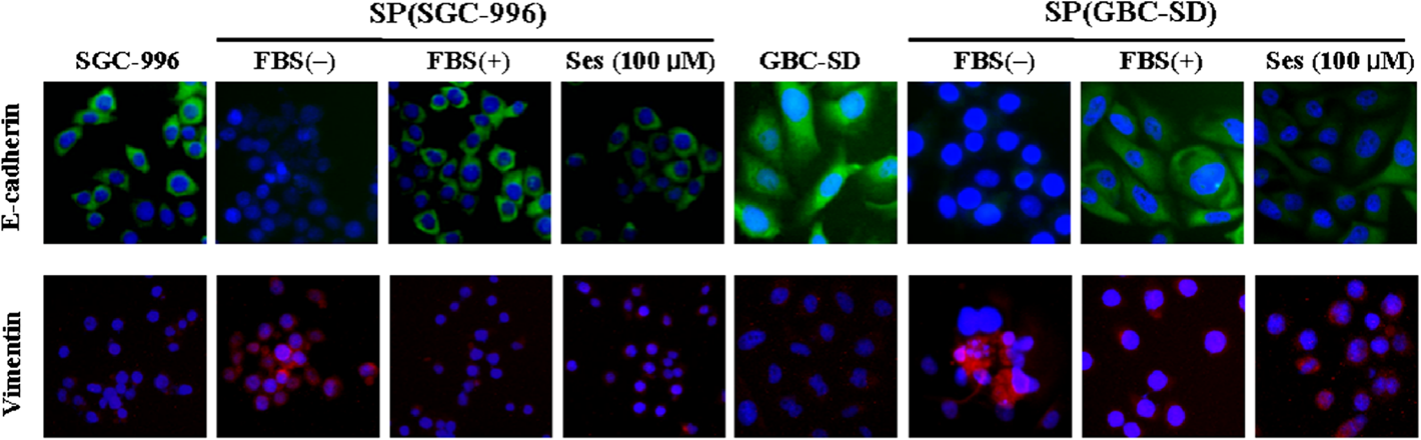
**Immunofluorescence staining for E-cadherin and Vimentin.** The expression of Vimentin and E-cadherin in SGC-996 and GBC-SD cells, SP cells in the absence or presence of the FBS, and SP cells treatment with 100 μM sesamin for 7 days.

The protein expression of p-Stat3, nuclear NF-κB, Vimentin and Twist detected by Western blot analysis was elevated in SP cells from SGC-996 and GBC-SD compared to non-SP cells. However, the expression of E-cadherin was just the opposite (Figure 3A). The mRNA and protein levels of IL-6 were also enhanced in SP cells compared to non-SP cells (Figure 3B and C). Immunofluorescence staining showed that SP cells harbored enhanced expressin of Vimentin and decreased expression of E-cadherin compared to parental cells (Figure 4). We also demonstrated that the expression level of Vimentin was decreased while E-cadherin was increased in SP cells under differentiating conditions (DMEM supplemented with 10% FBS in the absence of growth factors) (Figure 4).

### Sesamin effectively reduced the SP cells population

**Figure 5:**
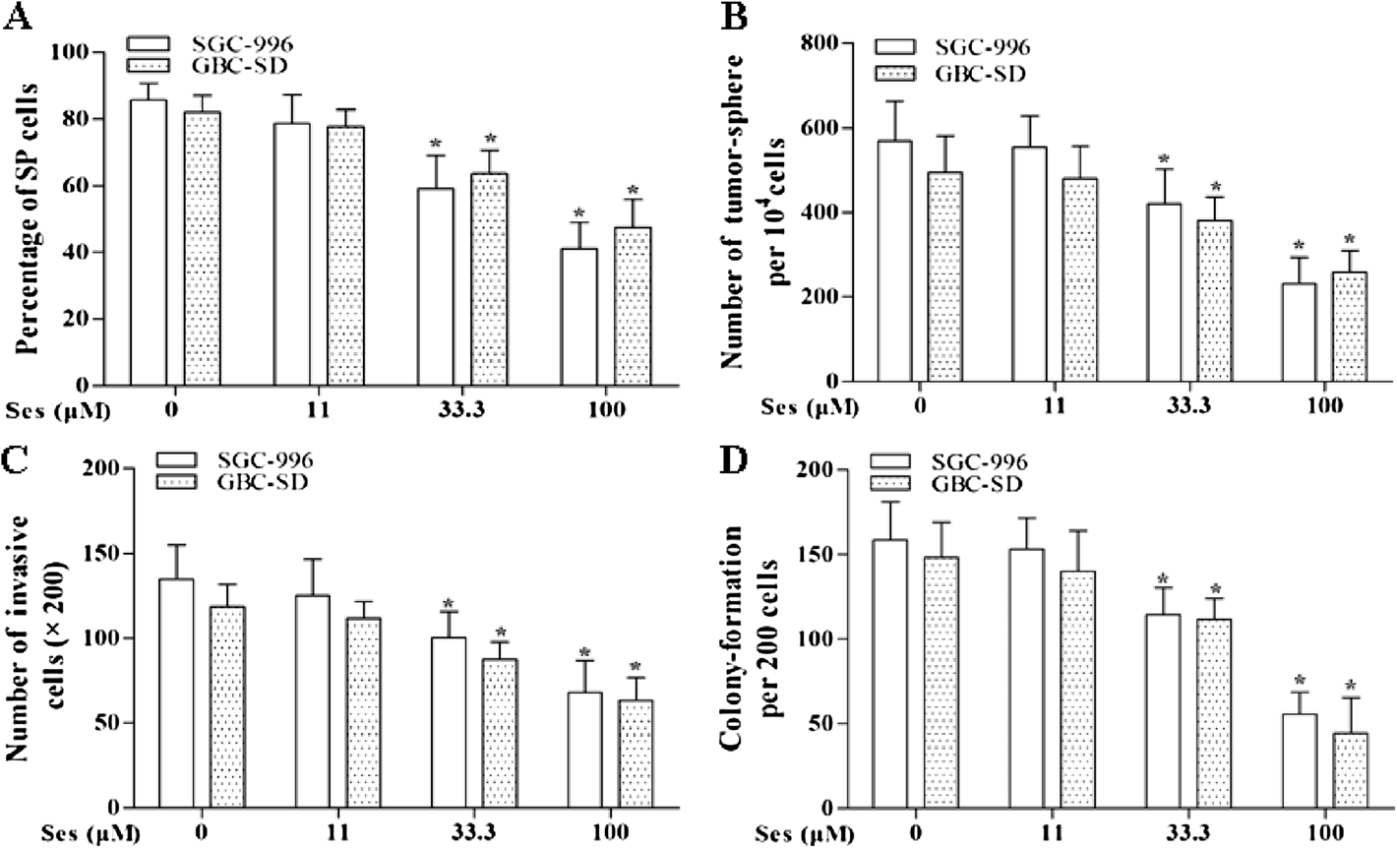
**Sesamin exerted its effects on SP cells.** Sesamin significantly decreased SP cells fraction in a dose-dependent manner **(A)**. Sesamin reduced the tumor-sphere formation ability of SP cells **(B)**. Sesamin reduced the invasion capacity of CSCs **(C)**. Sesamin inhibited colony formation of SP cells from SGC-996 and GBC-SD **(D)**. *p < 0.05 *vs.* control group. Ses: sesamin.

We measured the SP cells fraction after exposing to sesamin for 7 days under a non-differentiation condition. The results showed that sesamin significantly reduced the SP cells fraction in a dose-dependent manner, 100 μM sesamin diminished the SP cells fraction from 86%, 82% to 41%, 48% for SGC-996 and GBC-SD at day 7, respectively (Figure 5A).

### Sesamin modified stem cell-like features of SP cells

As shown in Figure 5B, sesamin markedly decreased the tumor-sphere formation ability of SP cells. Sesamin (100 μM) reduced the number of tumor-spheres by as much as 59%, 51% for SGC-996 and GBC-SD respectively. Sesamin also obviously attenuated the colony formation and invasion capacities of SP cells (Figure 5C and D).

### Sesamin inhibited SP cells growth

**Figure 6:**
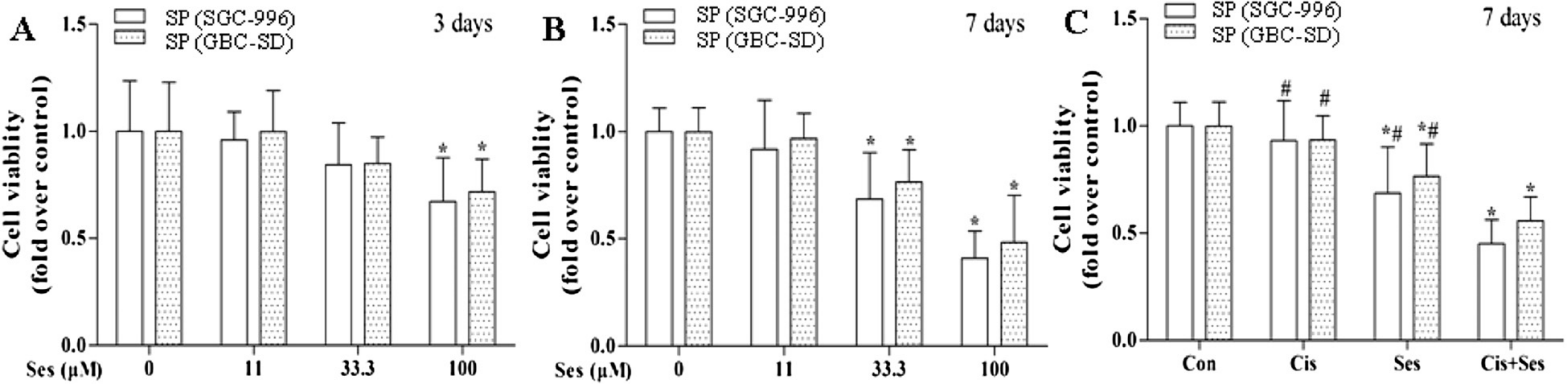
**Sesamin suppressed SP cells proliferation and enhanced the chemotherapeutic effect of cisplatin (Cis).** Sesamin effectively inhibited the viability of SP cells in a dose- and time-dependent manner **(A** and **B)**. Sesamin manifested its chemo-sensitization effects on SP cells from both SGC-996 and GBC-SD **(C)**. *p < 0.05 *vs.* control group. ^#^*p* < 0.05 *vs.* Cis + Ses combined treatment group. Ses: sesamin.

After treatment for 3 and 7 days, cell viability was assayed utilizing the CCK-8 method. Our data revealed that sesamin effectively inhibited the viabilities of SP cells in a dose- and time-dependent manner. SP cells viabilities were obviously decreased by treatment with sesamin (100 μM) to the extent of 41%, 48% for SGC-996 and GBC-SD at day 7, respectively (Figure 6A and B).

### Sesamin sensitized SP cells to the chemotherapeutic agent cisplatin

To explore the chemo-sensitization effect of sesamin, SP cells were treated with sesamin alone (33.3 μM), cisplatin alone (4 μM), sesamin plus cisplatin (33.3 plus 4 μM) for 7 days. Sesamin enhanced the chemotherapeutic effect of cisplatin on SP cells from both SGC-996 and GBC-SD (Figure 6C).

### Molecular mechanisms of sesamin targeting SP cells

**Figure 7:**
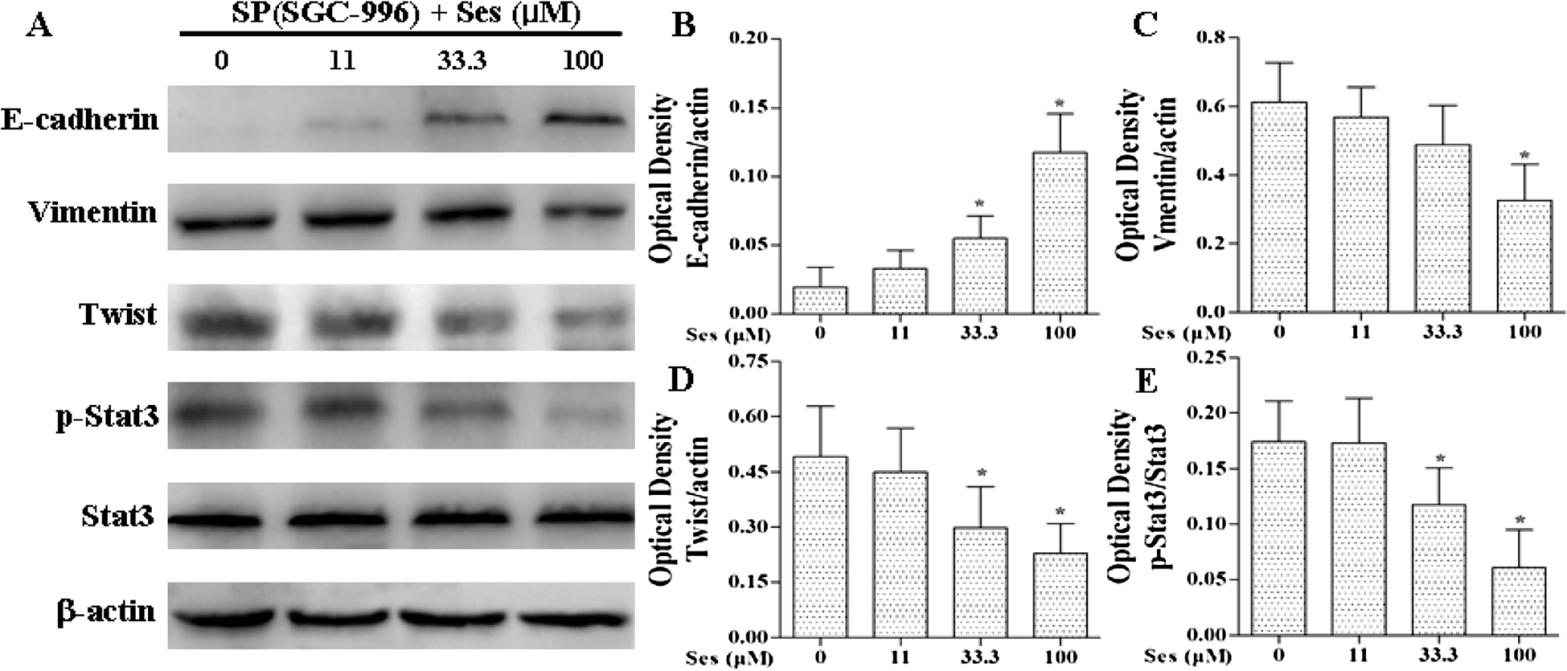
**Effects of sesamin on the protein expression of E-cadherin, Vimentin, Twist, Stat3 and p-Stat3 in SP cells from SGC-996 by Western blotting.** SP cells from SGC-996 were treated sesamin for 7 days. Panels show representative bands **(A)** and histograms represent optical density values normalized to the corresponding *β*-actin **(B-D)** or total Stat3 **(E)**. *p < 0.05 *vs.* control group. Ses: sesamin.

**Figure 8:**
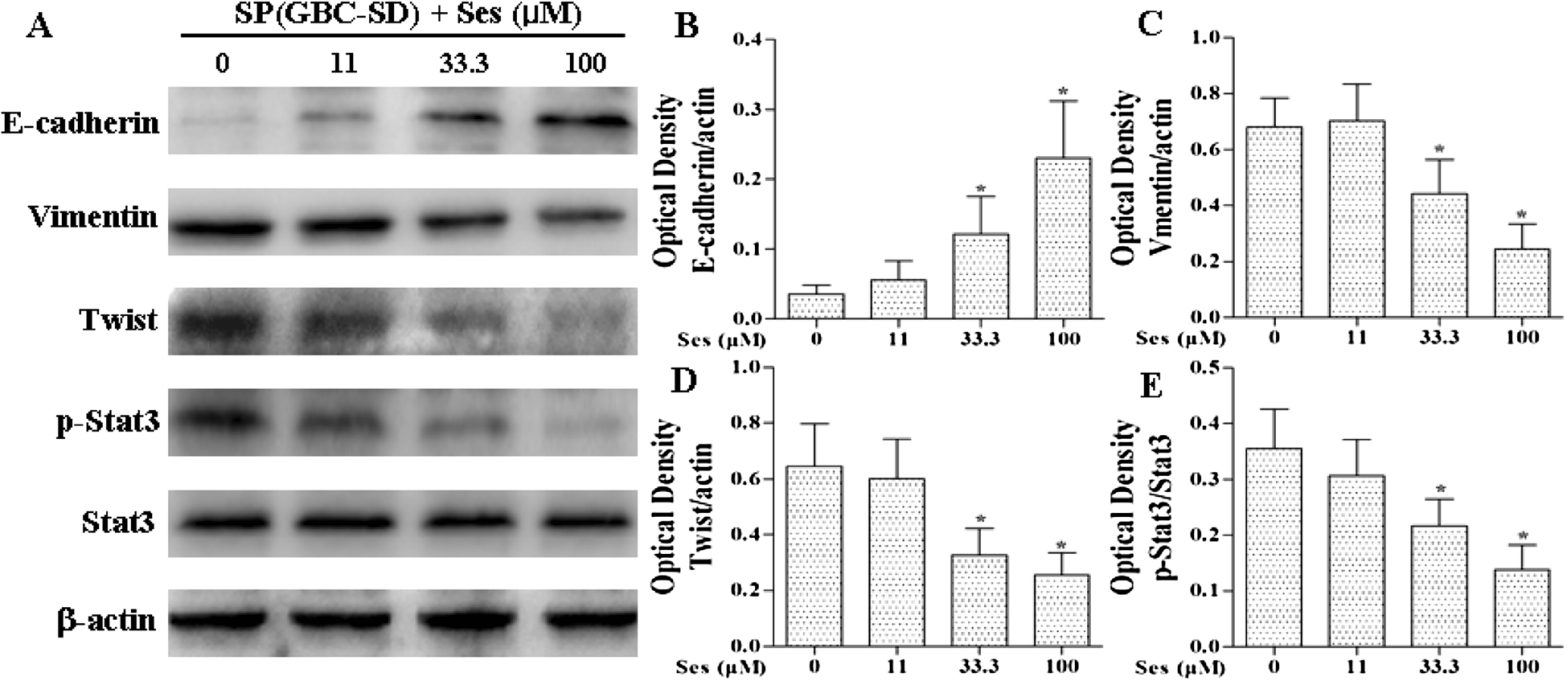
**Effects of sesamin on the protein expression of E-cadherin, Vimentin, Twist, Stat3 and p-Stat3 in SP cells from GBC-SD by Western blotting.** SP cells from GBC-SD were treated sesamin for 7 days. Panels show representative bands **(A)** and histograms represent optical density values normalized to the corresponding *β*-actin **(B-D)** or total Stat3 **(E)**. *p < 0.05 *vs.* control group. Ses: sesamin.

**Figure 9:**
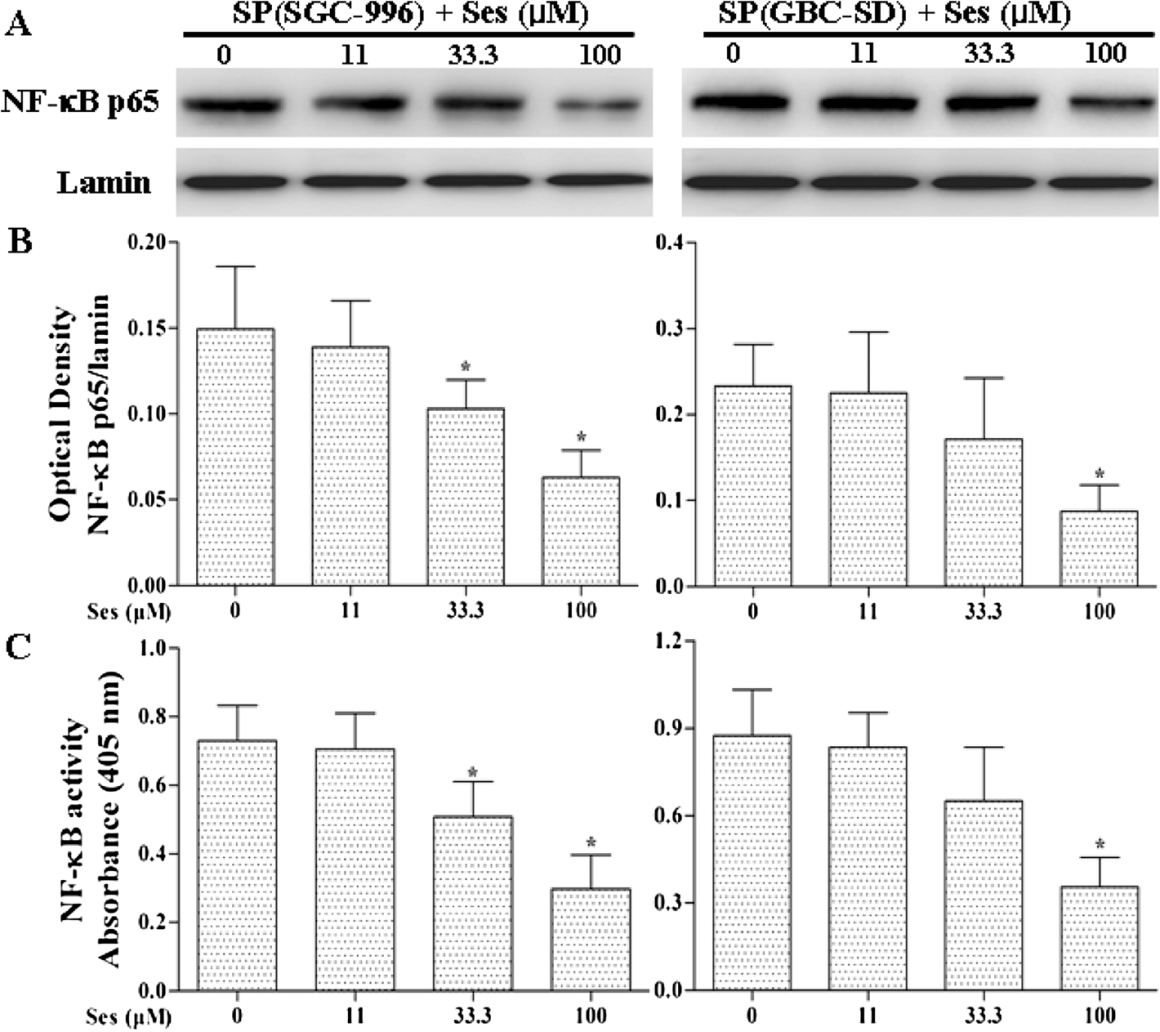
**Effects of sesamin on the protein expression and activity of nuclear NF-κB in SP cells.** Panels show representative bands **(A)**. Histograms represent optical density values normalized to the corresponding lamin **(B)**, and nuclear NF-κB activity **(C)**. *p < 0.05 *vs.* control group. Ses: sesamin.

**Figure 10:**
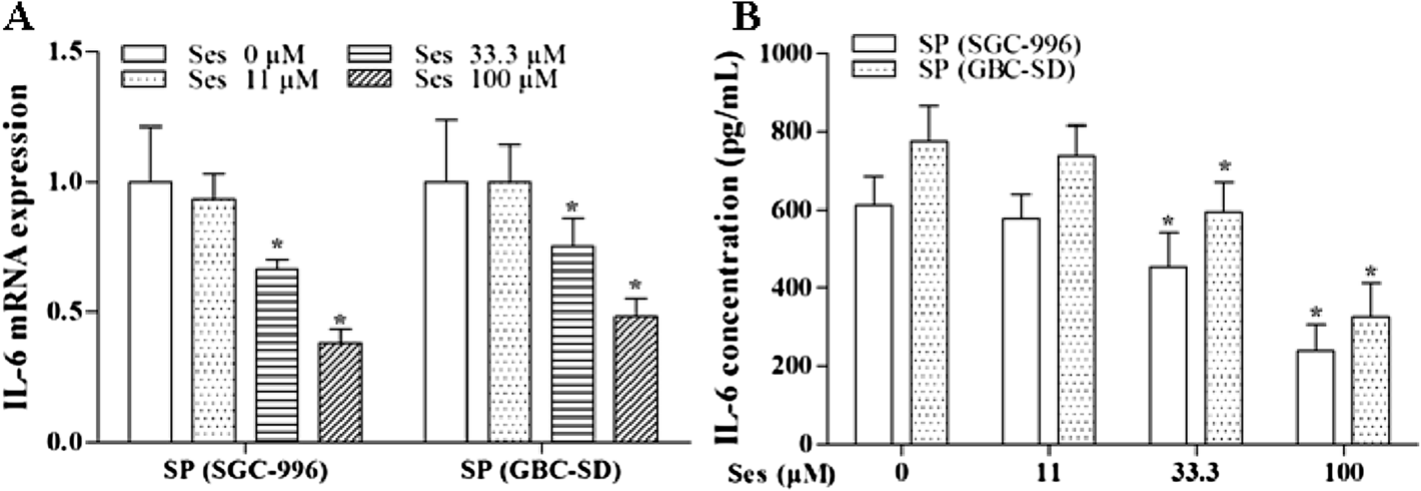
**Effects of sesamin on the mRNA expression and protein level of IL-6 in SP cells.** Histograms represent IL-6 mRNA expression **(A)** and protein level **(B)**. *p < 0.05 *vs.* control group. Ses: sesamin.

As shown in Figures 4, 7 and 8, sesamin enhanced the protein expression of E-cadherin (an epithelial marker), and decreased Vimentin (a mesenchymal marker) and Twist protein expression. We further examined the effects of sesamin on the NF-κB-IL-6-Stat3 pathway. Treatment with sesamin significantly decreased the protein expression of nuclear p-Stat3 and NF-κB as well as nuclear NF-κB activity (Figure 7A and E; Figure 8A and E; Figure 9). As shown in Figure 10, the mRNA expression and protein level of IL-6 were markedly reduced after treatment with sesamin for 7 days.

### Sesamin reduced SP cells-derived tumor growth *in vivo*

**Figure 11:**
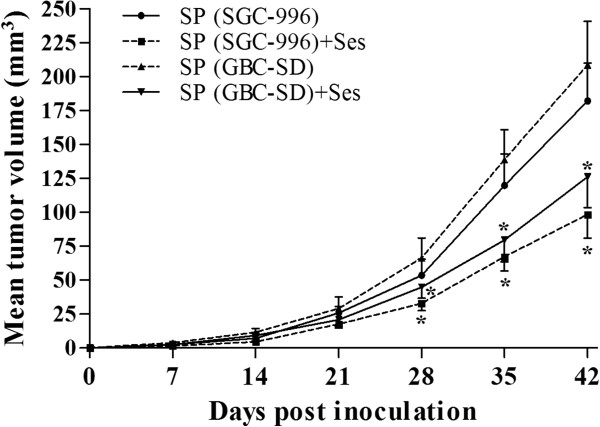
**Effects of sesamin on SP cells-derived tumor growth *in vivo*.** After pretreated with sesamin (100 μM) for 7 days, 1 × 10^5^ SP cells from both SGC-996 and GBC-SD were injected subcutaneously into 6-week-old nude mice (n = 6 each group). Tumor volumes were measured weekly. *p < 0.05 *vs.* control group. Ses: sesamin.

We assessed the tumor-inhibition effects by pretreated SP cells with sesamin (100 μM) alone for 7 days before injection. Difference in tumor volumes between the sesamin pretreatment group and the control group became significant since 28 days post inocubation. The results illustrated that sesamin significantly inhibited SP cells-derived tumor growth *in vivo* (Figure 11).

## Discussion

There is growing evidence that many human cancers are actually driven and maintained by a population of cells with stem-like properties. In addition to mediating tumor invasion, metastasis and recurrence, the comparative resistance of CSCs to conventional chemotherapeutic agents and radiation therapies may contribute to treatment resistance [22]. Thus, it is important to develop new therapies targeting CSCs. The CSCs have been identified and isolated on the basis of their expression of specific combinations of molecules, e.g., CD133, CD44, ATP-binding cassette proteins and aldehyde dehydrogenase [23]. In this study, we enriched the SP cells via the Hoechst dye assay. Our results showed that SP cells of GBC exhibited the stem cell-like abilities such as self-renewal, multi-differentiation, tumor initiation and chemo-resistance.

EMT is a well-coordinated developmental programme that has a very important role in the development of the mesoderm from the epithelium during embryogenesis [24,25]. Induction of EMT via down-regulating E-cadherin generates a stem cell phenotype, which contributes to higher invasive and metastatic abilities [21,26]. Consistent with aforementioned reports, our results revealed that the mesenchymal marker Vimentin was expressed at higher levels in SP cells while non-SP cells were with enhanced expression of the epithelial marker E-cadherin (Figure 3A). In other words, SP cells display a mesenchymal phenotype while non-SP cells an epithelial phenotype.

Plant-derived agents are now widely used in cancer therapy as supplemental or adjuvant agents. Food-derived agent, sesamin with its antitumor effects has drawn our attention [12-16]. In our study, we demonstrated that sesamin reduced the SP cells fraction and viability in a dose-dependent manner. Two explanations, either elimination [27,28] or differentiation [29,30] of SP cells may account for this phenomenon. The majority of the SP cells (more than 83%) were viable at this situation (after treatment with sesamin), however, as determined by trypan blue exclusion (data not shown). In fact, Wanachewin *et al*. [31] reported that sesamin has the ability to trigger osteoblast differentiation by activation of the p38 and ERK MAPK signaling pathway. In this study, we obtained epithelial differentiation of SP cells without shifting the cells to standard differentiating conditions (add 10% FBS in medium) but rather by maintaining the cells under non-differentiation conditions (which preserves *in vitro* their stem-like properties) in the presence of sesamin. Therefore, the decreased SP cells population may be attributed to the differentiation of SP cells.The hypothesis could be demonstrated by the following results: First, after exposure to sesamin for 7 days, the single-cell suspensions derived from primary tumor-spheres formed significantly fewer and smaller secondary tumor-spheres. Second, the differentiated cells achieved by shifting SP cells to differentiating conditions expressed the epithelial marker E-cadherin (Figure 4). Treatment with sesamin also resulted in the up-regulation of E-cadherin and down-regulation of Vimentin in SP cells, which was similar to the process of mesenchymal-epithelial transition. Third, sesamin modified the stem cell-like features. The sesamin-induced differentiation of SP cells was associated with a loss of properties that are considered the hallmarks of SP cells, such as the ability to form colonies, invade and to develop tumors in nude mice. Finally, we found that SP cells were more sensitive to a GBC therapy agent (cisplatin) after treatment with sesamin. This finding suggests that epithelial differentiation may sensitize SP cells to cytotoxic stimuli. In general terms, these results indicate that sesamin induces the differentiation of cancer stem-like SP cells from GBC.

EMT is mediated by the activation of transcription factors such as Twist, Snail and so on [32]. Ectopic expression of Twist results in down-regulation of E-cadherin (an epithelial marker), up-regulation of Vimentin (a mesenchymal marker) and expansion of the CSC population [33]. Cheng *et al*. [34] demonstrated that IL-6, Stat3 and Twist form a functional signaling axis to regulate pivotal oncogenic properties of cancer cells. Iliopoulos *et al*. [18] reported that the NF-κB-IL-6-Stat3 axis plays a vital role in the maintenance of CSCs. Taken together, it could be assumed that there exists a NF-κB-IL-6-Stat3-Twist axis in CSCs and it links the EMT programme and CSCs. In our study, the protein expression of Twist was much higher in cancer stem-like SP cells than in non-SP cells. We also demonstrated that the expression levels of nuclear NF-κB, IL-6 and p-Stat3 were enhanced in SP cells compared to non-SP cells.

In the meantime, interfering with this axis utilizing the NF-κB inhibitor [21,34], the IL-6 receptor antibody [21,35], the Stat3 inhibitor [21,30,36] or down-regulating the expression of Twist [26] reduces the CSC population and results in inhibition of tumor growth and metastasis. Generally, the NF-κB-IL-6-Stat3-Twist axis is vital for the maintenance of CSCs and association with the EMT process. Breaking this signal pathway may contribute to the epithelial differentiation of CSCs.

Aki *et al.*[13] and Lee *et al.*[37] reported that sesamin decreases the protein expression of NF-κB in breast cancer. Harikumar *et al.*[12] demonstrated that sesamin down-regulates constitutive and inducible NF-κB activation and expression in human chronic myeloid leukemia cell lines. In addition, Jeng *et al.*[17] showed that sesamin significantly inhibits IL-6 mRNA expression and protein level, and reduces nuclear NF-κB activity in microglial cells. In accordance with these reports, we found that sesamin also decreased IL-6 mRNA expression and protein level as well as nuclear NF-κB activity and protein expression in SP cells. Moreover, down-regulated protein expression of p-Stat3 and Twist was also observed in SP cells in a dose-dependent manner after treatment with sesamin for 7 days. These results suggest that the epithelial differentiation effect of sesamin is associated with the broken of NF-κB-IL-6-Stat3-Twist axis.

## Conclusions

In summary, our study suggests that sesamin directs the epithelial differentiation of cancer stem-like SP cells from GBC. These effects are associated with the attenuation of NF-κB-IL-6-Stat3-Twist signal pathway. It meets the criteria for treatment strategies: Depletion of the CSC pool and generation of differentiated nontumorigenic cells with increased sensitivity to chemotherapy.

## Competing interests

The authors declare that they have no competing interest.

## Authors’ contributions

Conceived and designed the experiments: XK, MZM, QS, ZWQ and JRY. Performed the experiments: XK, MZM, YZ, MZW, LQG, JXZ and GDW. Analyzed the data: XK, MZM, YZ and WG. Wrote the paper: XK, MZM, QS and JRY. All authors read and approved the final manuscript.

## Pre-publication history

The pre-publication history for this paper can be accessed here:

http://www.biomedcentral.com/1472-6882/14/254/prepub
